# Infantile osteoarticular tuberculosis misdiagnosed as Bacillus Calmette-Guerin related osteomyelitis

**DOI:** 10.1051/sicotj/2015021

**Published:** 2015-07-21

**Authors:** Noppachart Limpaphayom, Phatcharapa Osateerakun, Apiradee Theamboonlers, Sumeth Korkong, Yong Poovorawan

**Affiliations:** 1 Department of Orthopaedics, Faculty of Medicine, Chulalongkorn University 10330 Bangkok Thailand; 2 Department of Orthopaedics, King Chulalongkorn Memorial Hospital, Thai Red Cross Society 10330 Bangkok Thailand; 3 Center of Excellence in Clinical Virology, Department of Pediatrics, Faculty of Medicine, Chulalongkorn University 10330 Bangkok Thailand

**Keywords:** Infant, *Mycobacterium bovis*, *Mycobacterium tuberculosis*, Osteomyelitis, Debridement

## Abstract

Tuberculosis, a re-emerging public health problem, is uncommon in infancy. Two healthy completely immunized infants presenting with manifestations compatible with osteoarticular infection required surgical debridement. The cultures of the specimens were positive for *M. tuberculosis* (MTB) complex comprised multiple subspecies. One case was misdiagnosed as a Bacillus Calmette-Guerin (BCG) related osteomyelitis by a polymerase chain reaction (PCR) based on detection of genes at the region of difference 1. Genome extraction and PCR using the *rimM* gene and sequences analysis against MTB and BCG control samples confirmed that both specimens were infected by *M. tuberculosis*. The lesions were successfully healed within one year. Surgical debridement of suspected lesions is warranted in infants as a definitive treatment and to obtain tissues for further evaluation. Microbiological cultures only confirm nonspecific MTB complex infection. PCR kits may yield a false positive result. Identification of the pathogen by DNA extraction and sequence analysis should be recommended.

The *Mycobacterium tuberculosis* (MTB) complex infection, a re-emerging public health problem, is uncommon in infancy [1, 2]. The Bacillus Calmette-Guerin (BCG) vaccine, a live attenuated *Mycobacterium bovis*, provides significant benefits of disease prevention. However, adverse effects of BCG vaccination have been reported recently [3, 4]. Clinical manifestations of the MTB complex, e.g. *M. tuberculosis*, *M. bovis*, and *M. africanum*, osteomyelitis are similar and a variety of laboratory tests have been used to identify the pathogen. The recommended treatments are also variable in this age group [2, 4].

We report *M. tuberculosis* osteoarticular infection in infants who were successfully treated by surgical debridement. The specimen in one case was misdiagnosed as *M. bovis* BCG infection using a commercial polymerase chain reaction (PCR) kit. The organism was later confirmed by PCR for the *rimM* gene and sequence analysis.

## Case report

**Figure 1.**
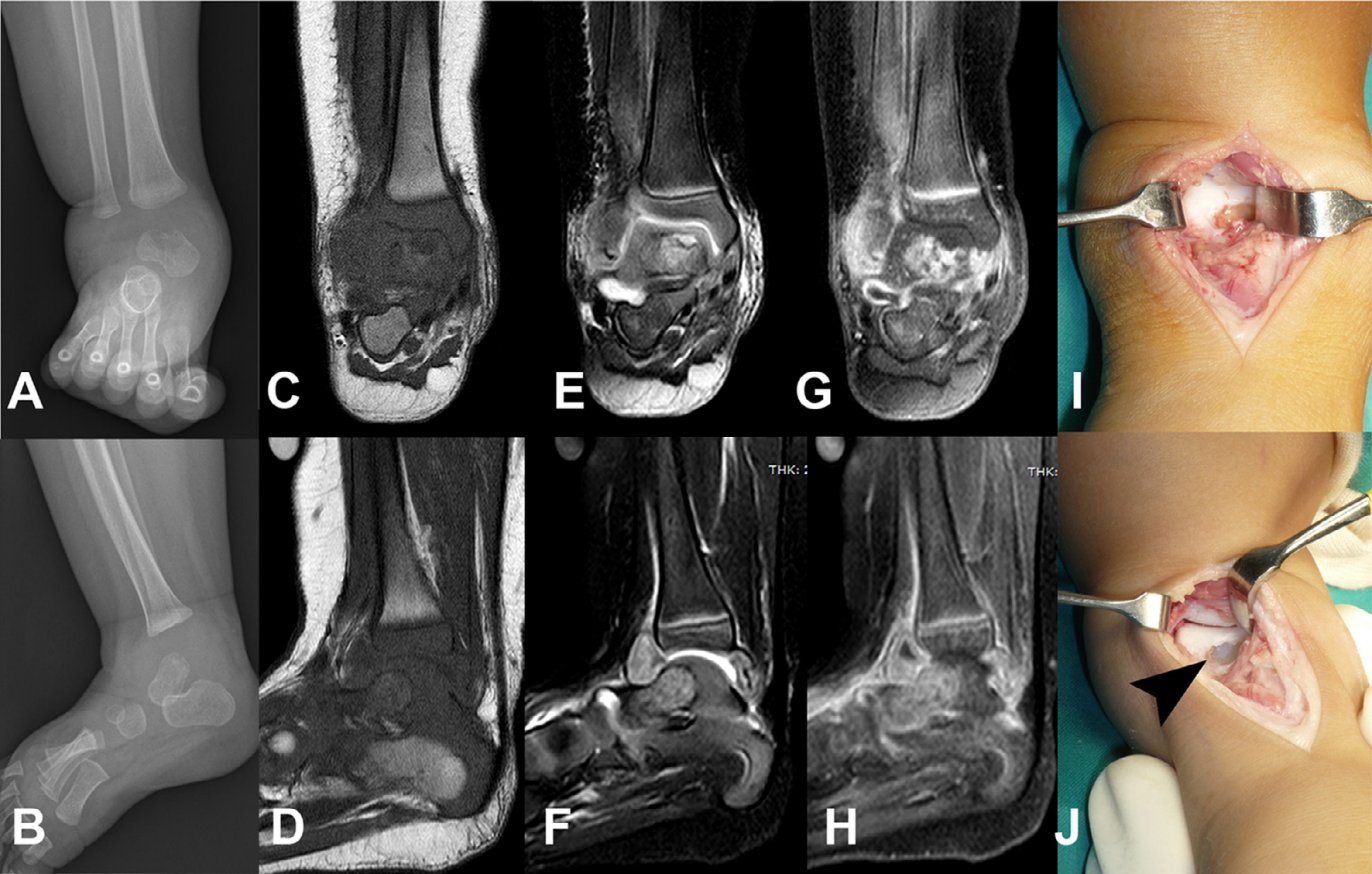
Anteroposterior (A) and lateral (B) radiographs of the right ankle of patient 1 are shown along with magnetic resonance imaging images in T1 weighted (C, D), T2 weighted (E, F), and gadolinium-enhanced (G, H). Intraoperative finding before (I) and after (J) debridement is demonstrated. The arrowhead represents an osteolytic lesion at the talus.

**Figure 2.**
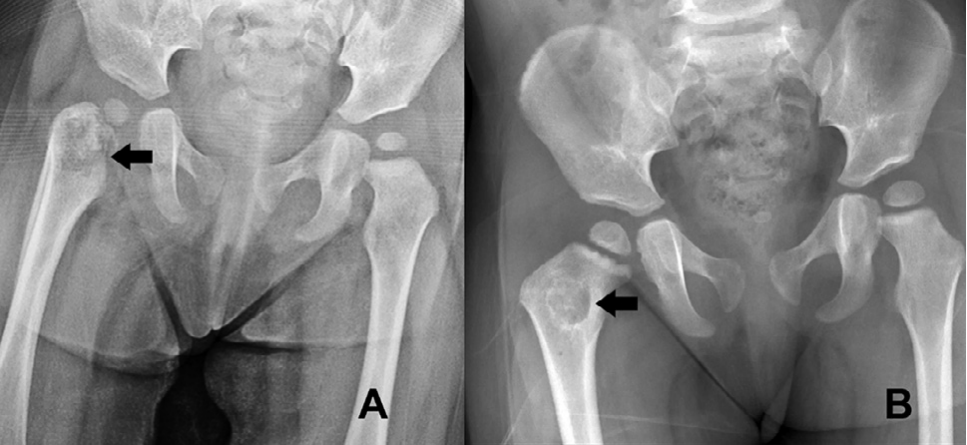
Radiographs of the hips of patient 2 are shown before surgery (A) and at 12-month follow-up visit (B). The arrows represent an osteolytic lesion at the right proximal femur.

Two infants presented with the chief complaint of a painful swollen and diminished range of movement of an extremity with an osteolytic lesion at the metaphysis adjacent to the involved joint. Due to the presentations, open debridement and curettage were performed. Clinical details are shown in Table 1. The radiographic studies and intraoperative findings of patient 1 are shown in Figures 1A–1J. The radiographic study of patient 2 is shown in Figure 2A. The source cases of *M. tuberculosis* infection in the respective family could not be identified. The study was approved by the Institutional Review Board Committee of the Faculty of Medicine, Chulalongkorn University, Thailand (COE 017/2013).

**Table 1.** Demographic and laboratory data of both patients.

Patient data	Patient 1	Patient 2	Reference range
*Gender/age* (months)	Female/6	Female/12	
*Location/duration*	Right talus and ankle joint/1 week	Right proximal femur and hip joint/2 weeks	
*Physical examination*			
Body temperature (°C)	37	37	
BCG scar	Left shoulder	Left gluteal	
Body weight (kg)	6.6	9.3	
Height (cm)	68	73	
PPD skin test (mm)	18	15	
			
*Laboratory findings*			
CBC			
Hb (g/L)	110.0	127.0	120.0–150.0
Hct (proportion of 1.0)	0.33	0.39	0.36–0.45
WBC (×10^9^/L)	9.83	17.48	4.5–11.0
Neutrophils (proportion of 1.0)	0.40	0.24	0.40–0.71
Lymphocytes (proportion of 1.0)	0.49	0.70	0.22–0.43
Monocytes (proportion of 1.0)	0.09	0.04	0.0–0.07
Eosinophils (proportion of 1.0)	0.03	0.01	0.0–0.04
Basophils (proportion of 1.0)	0.004	0.002	0.0–0.02
ESR (mm/h)	54	9	0–28
C-Reactive protein (mmol/L)	331.4	55.24	<47.62
Chest X-ray	Normal	Normal	

BCG, Bacillus Calmette-Guerin; PPD, purified protein derivative; CBC, complete blood count; Hb, hemoglobin; Hct, hematocrit; WBC, white blood cell; ESR, erythrocyte sedimentation rate.

**Figure 3.**
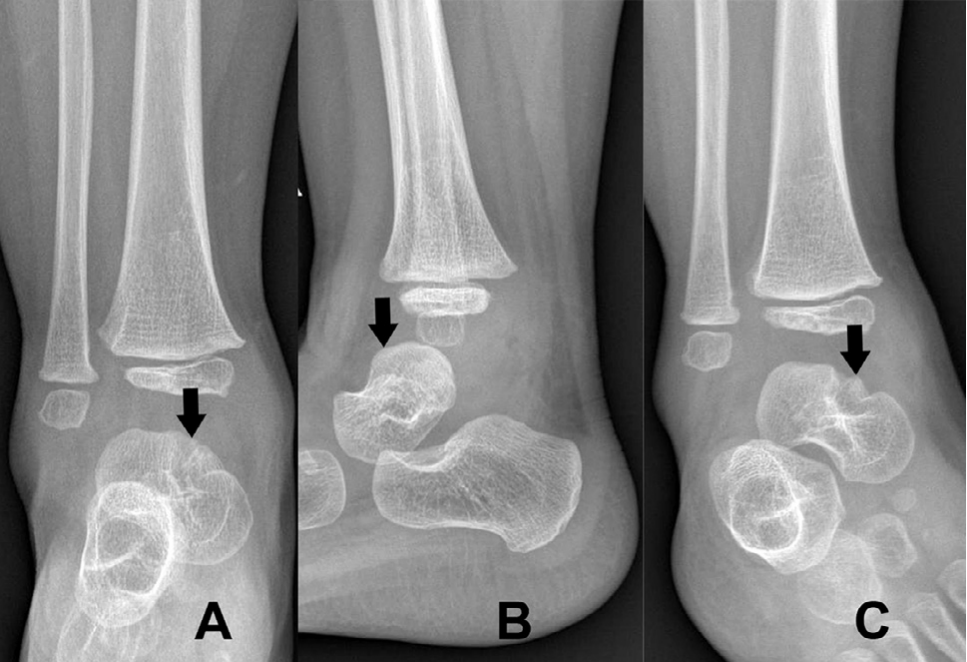
Radiographs of patient 1 at 12-month follow-up are demonstrated. The arrows represent an osteolytic lesion at the talus.

Microbiologic profiles are presented in Table 2. The specimens from both infants had a positive culture for MTB complex. The PCR for BCG using Seeplex^®^ MTB/BCG kit (Seegene, Seoul, Korea) to detect the genes in the region of difference 1 (RD1) was done in patient 2 after a subsequent case of suspected MTB complex infection was presented in an infant who was healthy and showed a positive result for a BCG. *M. tuberculosis* infection was subsequently confirmed by DNA extraction and sequence analysis. A standard 12-month course of anti-tuberculosis regimen including isoniazid, rifampicin, ethambutol, and pyrazinamide was prescribed. Both patients resumed full-weight bearing ability after 3 months. At 12-month follow-up, both infants demonstrate a satisfactory healing radiographically and the radiographs are shown in Figure 2B for patient 1 and Figure 3 for patient 2, respectively.

**Table 2.** Results of bacteriological investigations of both patients.

	Patient 1	Patient 2
*Surgical specimen*		
Aerobic culture	No growth	No growth
Mycobacterium culture	*Positive* for *Mycobacterium*	*Positive* for *Mycobacterium*
	*tuberculosis* complex	*tuberculosis* complex
Gram stain	No organism	No organism
AFB stain	Positive for AFB stain	Negative for AFB stain
		
Gastric lavage culture	No growth	No growth
Hemoculture	No growth	No growth
Pathological examination	Granulomatous osteomyelitis	Granulomatous osteomyelitis
PCR for BCG by commercial kit	N/A	*Positive*

AFB, acid-fast bacilli; PCR, polymerase chain reaction; BCG, Bacillus Calmette-Guerin; N/A, not available.

### Detection of MTB and BCG

**Figure 4.**
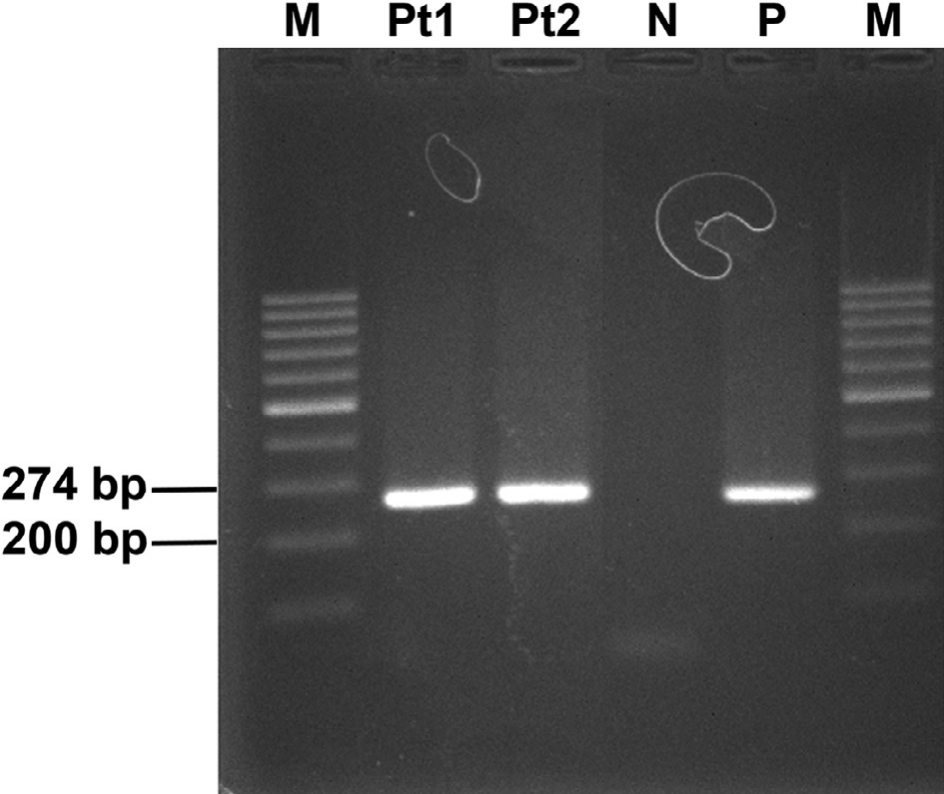
Electrophoresis of the polymerase chain reaction products generated a band of 274 bp in good agreement with the expected size. M,100 bp marker; Pt1, sample from patient 1; Pt2, sample from patient 2; N, negative control; and P, positive control.

The PCR was performed using F3 5′ CTAAGGGGCCTTTTGACGG 3′ as a sense primer and B3 5′ CACACTTCGGTGACGACAC 3′ as an antisense primer and was subjected to gel electrophoresis (Figure 4). The amplified product (274 bp) was excised. The nucleotide sequences were analyzed using the BCG vaccine (Queen Saovabha Memorial Institute, Thailand) and the *M. tuberculosis* culture was used as a positive control.

**Figure 5.**
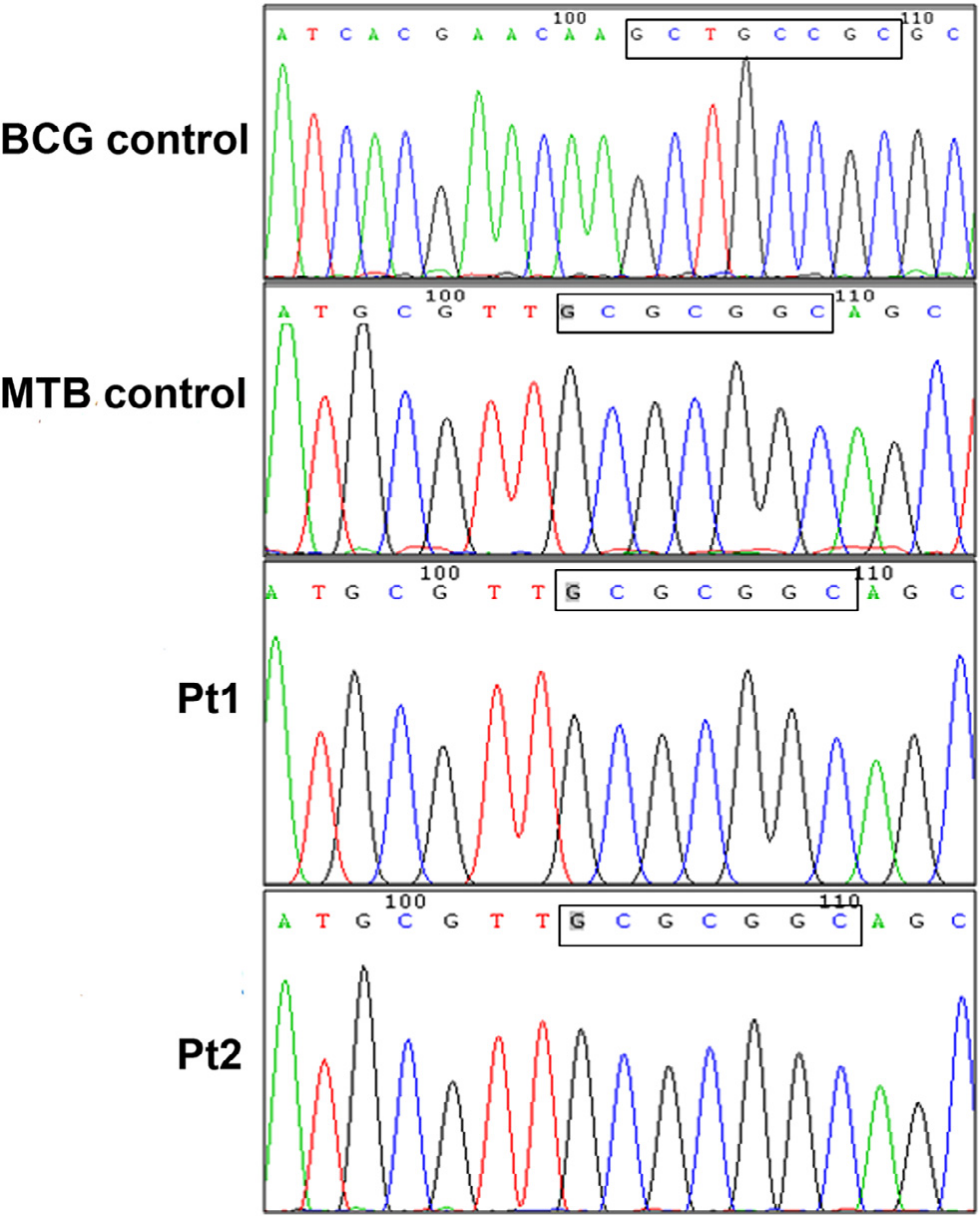
Chromatograms of nucleotide sequences of the two positive samples compared with *Mycobacterium tuberculosis* and Bacillus Calmette-Guerin control samples showing sequences identical with *Mycobacterium tuberculosis*. MTB, *Mycobacterium tuberculosis*; BCG, Bacillus Calmette-Guerin; Pt1, sample from patient 1; Pt2, sample from patient 2.

Nucleotide sequences were compared with the *rimM* gene of the reference strain of *M. Tuberculosis* H37Rv (Gene ID: 887188). The alignment between the studied samples and the strain of *M. Tuberculosis* H37Rv in partial *rimM* gene showed 100% identity. The sequences were subjected to BLAST analysis and displayed 99% sequences homology with the strain obtained from *M. tuberculosis* EAI5 isolated in Mumbai, India. Chromatograms of nucleotide sequences of the two samples compared with those of *M. tuberculosis* and *M. bovis* BCG control sequences showed the identical sequences with *M. tuberculosis* (Figure 5).

## Discussion

*M. tuberculosis* osteoarticular infection in children aged less than 12 months is rare [1, 2]. Due to the high prevalence of *M. tuberculosis* in the region, the diagnosis is high on the list when evaluating a child with bone and joint pain [1, 4]. The clinicians require a high index of suspicion, should be familiar with the clinical presentations and aware of other possible causative organisms [4]. The BCG vaccine consisted of at least six BCG substrains and it is variable among countries [5]. The BCG Tokyo-172 strain was utilized in several Asian countries including South Korea, Taiwan, and Thailand, while others, e.g. China and Hong Kong, utilized the Danish strain [1, 3–5]. Osteoarticular infection caused by the BCG vaccine may occur [3, 4].

Clinically, *M. tuberculosis* and *M. bovis* BCG-related osteomyelitis is indistinguishable [2, 3]. Painful swelling of the extremity, decreasing range of motion of an affected joint in the early stage, and no or low grade fever raises a possibility of osteoarticular infection. There has been an increasing awareness of BCG Tokyo-172 strain associated complications which raised concerns regarding its safety and the vaccine is under a surveillance program in some countries [3]. Hematogenous spread would be the most likely explanation since the affected extremity was not limited to the site of the BCG administration [4]. This could be a serious threat to the reputation of the national vaccination program.

The radiographic appearances are usually nonspecific for both organisms including poorly demarcated cystic lesions and minimal sclerotic reactions at the metaphysis or the epiphysis [4]. The joint space is preserved in the early stage and gradually damaged by indirect subchondral erosion and pannus formation [2]. Synovitis, joint effusion, and epiphyseal and metaphyseal involvement are common findings shown on MRI images. Additionally, an MRI is a useful investigation for a preoperative planning [4]. Pathological examination usually shows nonspecific granulomatous osteomyelitis. Moreover, an MTB complex culture comprises multiple organisms. Other pathogens, e.g. *M. bovis* BCG, should be tested for when an apparent MTB complex infection occurs in an infant with a successful immunization confirmed by a visible BCG scar and a positive tuberculin skin test but unidentifiable *M. tuberculosis* source cases [4].

Although anti-tuberculosis chemotherapies remain a principal treatment, pyrazinamide, a recommended first line treatment of *M. tuberculosis* infection, is not effective against an *M. bovis*, thus early differentiation between the two pathogens by a reliable test is crucial. While chemotherapeutic treatment without surgical debridement of the lesion was suggested, this could be challenging in a certain age group. The fine needle aspiration was found to yield a positive result in 1/3 to 2/3 of cases [4]. By contrast, our treatment is justified by presentations of infants, an age group where verbal communication is inadequate, and the high prevalence of *M. tuberculosis* [1, 2, 4]. Surgical debridement was recommended to improve the general condition of the infant and remove necrotic tissue. This allows chemotherapeutic agents to eradicate the MTB sequestrated in the osteolytic lesion and a specimen is obtained to ascertain a diagnosis in a patient from an area endemic for bacterial and fungal infections [2, 4]. Our experience showed that the osteolytic lesion healed satisfactorily at one year as shown on the radiograph.

The usefulness of the PCR method to differentiate between *M. tuberculosis* and BCG has been reported [3, 4]. Genes in the RD1 region produce proteins responsible for the virulence of *M. tuberculosis* and they are present in every strain of *M. tuberculosis* but by contrast, are absent in the BCG [5]. Our result demonstrates that a commercial PCR kit can potentially produce a false positive result. The *rimM* gene is the 16S rRNA-processing protein gene and the sequence is specific for *M. tuberculosis* and *M. bovis*. We choose to study genes in difference genomic region to avoid a false positive result and found that our samples contained either mycobacterium species which were later confirmed to be *M. tuberculosis* by sequence analysis. The most frequent explanation for false positive PCR results is sample contaminations; but this is very unlikely since the BCG is not a routine organism being tested. We recommend that the investigations should include a complete set of investigation for all possible organisms based on the incidence and geographical zone [2, 4].

In conclusion, prompt surgical debridement of an osteolytic lesion suspected of being an infection is warranted in an infant. Differentiation between *M. tuberculosis* and *M. bovis* BCG-related infection in those who have received BCG vaccination should be performed when an *M. tuberculosis* source is unidentifiable. A specimen culture is the gold standard for mycobacterial infection but provides only nonspecific result. The PCR kit could be used as a screening test for *M. bovis* BCG infection. DNA extraction and sequence analysis is more time-consuming but could be recommended as a confirmation method.

### Conflict of interest

The authors, NL, PO, AT, SK, and YP, certify that they have no financial conflict of interest in connection with this article. No benefits in any form have been received or will be received from a commercial party related directly or indirectly to the subject of this article.
